# Family Physician attitudes about prescribing using a drug formulary

**DOI:** 10.1186/1471-2296-10-69

**Published:** 2009-10-16

**Authors:** L Suzanne Suggs, Parminder Raina, Amiram Gafni, Susan Grant, Kevin Skilton, Aimei Fan, Karen Szala-Meneok

**Affiliations:** 1 Institute of Communication and Health, Facultyof Communication Sciences, University of Lugano, Switzerland; 2 Department of Clinical Epidemiology and Biostatistics, McMaster University, Hamilton, Ontario, Canada; 3 Evidence-based Practice Center, McMaster University, Hamilton, Ontario, Canada; 4 Merck Frosst Canada, Kirkland, Quebec, Canada; 5 Public Health Services, City of Hamilton, Hamilton, Ontario, Canada; 6 School of Rehabilitation Sciences, McMaster University, Hamilton, Ontario, Canada

## Abstract

**Background:**

Drug formularies have been created by third party payers to control prescription drug usage and manage costs. Physicians try to provide the best care for their patients. This research examines family physicians' attitudes regarding prescription reimbursement criteria, prescribing and advocacy for patients experiencing reimbursement barriers.

**Methods:**

Focus groups were used to collect qualitative data on family physicians' prescribing decisions related to drug reimbursement guidelines. Forty-eight family physicians from four Ontario cities participated. Ethics approval for this study was received from the Hamilton Health Sciences/Faculty of Health Sciences Research Ethics Board at McMaster University. Four clinical scenarios were used to situate and initiate focus group discussions about prescribing decisions. Open-ended questions were used to probe physicians' experiences and attitudes and responses were audio recorded. NVivo software was used to assist in data analysis.

**Results:**

Most physicians reported that drug reimbursement guidelines complicated their prescribing process and can require lengthy interpretation and advocacy for patients who require medication that is subject to reimbursement restrictions.

**Conclusion:**

Physicians do not generally see their role as being cost-containment monitors and observed that cumbersome reimbursement guidelines influence medication choice beyond the clinical needs of the patient, and produce unequal access to medication. They observed that frustration, discouragement, fatigue, and lack of appreciation can often contribute to family physicians' failure to advocate more for patients. Physicians argue cumbersome reimbursement regulations contribute to lower quality care and misuse of physicians' time increasing overall health care costs by adding unnecessary visits to family physicians, specialists, and emergency rooms.

## Background

In Canada and elsewhere, in recognition of the fact that resources for health care (including drugs) are constrained, formularies have been established to maximize the health of the population from available resources. With the rise of formularies, physicians grapple with issues such as: determining best therapeutic choices based on current evidence and clinical experience, and formulary rules controlling third party payers' costs [1-6].

Most research in this area has relied on quantitative approaches such as observational studies of prescription drug claims data[7,8] to provide information on what physicians prescribe, but are unable to provide insight into physicians' attitudes about the prescribing process. Qualitative methods are well suited to identify and explore the complex decision-making processes physicians experience when prescribing.

### The Ontario Drug Benefit Program (ODBP)

The Government of Ontario manages a publicly-funded prescription drug insurance plan for eligible beneficiaries. The Ministry of Health and Long-term Care (MOHLTC) provides coverage through the ODBP formulary for patients who are over 65 years or who receive social assistance. This formulary also applies to the ODBP's Trillium drug program to which individuals apply for coverage for prescription expenses in excess of a threshold amount relative to their income.

The MOHLTC established the formulary in 1974 in consultation with the ministry's external expert drug advisory committee, the Drug Quality and Therapeutics Committee (DQTC), that provides independent and specialized advice to the Minister of Health and the Drug Programs Branch on drug-related issues. The DQTC (now known as the Committee to Evaluate Drugs) monitors and evaluates the list of drugs available based on drug use patterns, experience, and current scientific knowledge monthly. For drug products to be eligible for consideration for listing in the formulary, a drug manufacturer must provide a complete submission in accordance with provincial drug legislation. A complete submission undergoes a thorough review using current therapeutic guidelines and evidence-based data[9].

In June of 2006, Royal Assent was granted *The Transparent Drug System for Patients Act, 2006 *that revised a number of elements of the publicly funded insurance program following a comprehensive program review. The MOHTLC's objectives for the ODBP reforms were: improving patient access to drugs, ensuring better value for money, promoting the appropriate use of drugs, rewarding innovations, and strengthening transparency and accountability[10].

This article describes the three levels of drug coverage and the associated administrative burden on physicians as they existed in 2002, prior to the reforms. Under the first ODBP level of coverage, physicians could prescribe general benefit (GB) drugs without consulting reimbursement criteria. When drugs are available from multiple manufacturers, however, the ODBP required pharmacists to dispense products with the lowest listed prices. The second level of coverage permitted physicians to prescribe Limited Use (LU) drugs. LU prescriptions required precise eligibility codes that justified LU drugs for treatment of predefined conditions under restricted circumstances. LU prescriptions required physicians to handwrite and sign prescriptions using special ministry-supplied prescription pads. The prescriptions were valid for one year from the signing date.

A third level of ODBP coverage applied to drugs not listed as benefits. Drugs may not be listed on the ODBP if the DQTC finds the evidence has not shown them to be effective or safe for the patient population that is covered by the Ontario public plan. These drugs may be made available under special circumstances i.e., "to treat conditions or diseases that would otherwise cause severe debilitating effects"[9] to patients who are unable to benefit from any of the drugs listed by the ODBP (GB or LU), or who do not meet the strict LU drug eligibility criteria[11]. For coverage to be granted for unlisted drugs, physicians had to carefully document a clinical description and therapeutic plan and other information for reimbursement under the Individual Clinical Review Mechanism (Section 8) of the ODBP. Medical experts contracted by the MOHTLC reviewed the request and advised physicians if coverage was approved. Under the regulations in place in 2002, coverage had to be renewed annually. A full description of this mechanism is available in Part VIII of the formulary[9].

The objective of this qualitative study was to examine family physicians' attitudes toward restrictions on prescription reimbursement through the ODBP and how this influences their prescribing practices. Physicians articulated that formulary requirements exacerbate existing tensions in clinical practice. This study, however, does not provide a definitive evaluation of the consequences of reimbursement criteria nor does it compare and contrast systems. Rather, it more fully explores the attitudes of physicians in relation to the public drug insurance program in Ontario as it was structured in 2002, and expands on physicians' sentiments to suggest that additional research is needed to determine how formulary management practices may impact patient outcomes[12].

## Methods

### Participants

Using purposive sampling, family physicians from four cities in Southern Ontario were recruited to participate in one of six focus group sessions in this exploratory qualitative study. In cooperation with the researchers, recruitment was conducted by Pollara, a national public and private research firm. For the purposes of this study, Pollara contacted physicians who met practice-type eligibility criteria with the intent to find a sample of Ontario family physicians. Physicians were drawn from larger and smaller cities (population range: 2.4 million to 74 thousand), and were in full-time family practice in either private practice, academic or non-academic hospital settings. Physicians recruited had active practice experience that ranged from ≤ 10 years to ≥ 10 years, and experience in caring for patients > 65 years of age.

A total of 51 physicians agreed to participate and 48 attended the sessions. Eighty percent of the physicians were in private practice with 10% of the remainder practicing in academic and non-academic hospital settings, respectively. Most of the physicians were male 69% (n = 33), which approximates Ontario's family physician gender ratio, 100% were in clinical practice more than 80% of their time, and 85% had been in practice for more than 10 years. Their practice populations included 10 - 75% of patients over the age of 65 (mean = 36%). Having experience prescribing for seniors is important given persons 65 years of age and over are the largest group of beneficiaries under the ODBP. A $200.00 (Canadian) incentive was provided. In this focus group research, sample size was estimated by aiming for 6-8 persons per group with a base of 3-4 groups or more until theoretical saturation and information richness was reached[13].

Ethics approval was received for this study from McMaster University's Hamilton Health Sciences/Faculty of Health Sciences Research Ethics Board (REB).

### Focus Groups

Focus group questions were developed and pre-tested by the investigators with five physicians who were representative of the study population. Six focus groups were conducted over a two week period in December 2002 with an average of eight physicians per focus group. Sessions averaged two hours in length and were led by a trained focus group facilitator.

At the start of each focus group, the facilitator explained the purpose of the session and asked physicians to sign a statement of informed consent. Once those were signed, physicians were presented with two of four possible clinical practice scenarios describing conditions commonly managed by family physicians (see Table 1). The patients in the scenarios were either ODBP beneficiaries or self-pay or private insurance beneficiaries. Medications that might be prescribed included those available as a GB or through LU or section 8 processes. The two scenarios were intended to situate the participants in a patient encounter leading to a prescribing decision; thus, they were asked to quietly read each scenario and write a mock prescription. The moderator then used semi-structured and open-ended questions to facilitate interaction and guide participants through a discussion of their decision-making process when prescribing and attitudes about their prescribing decisions.

**Table 1:** Clinical practice scenarios employed to situate focus group sessions

**Condition**	**Presentation**
**Asthma**	A 35 year old asthmatic woman has recently moved into subsidized housing where there are cats. One year ago she had a normal spirometry but has recently experienced coughing, and dyspnea with wheezing and disturbed sleep. She inhales budesonide daily and has increased her use of terbutaline with the recent increase in symptoms. She is reluctant to increase her us e of corticosteroids.
**Hypertension**	A 55 year old male has type 2 diabetes that is currently managed with diet and exercise. Physical exam, ECG, BMI, and renal function are normal. Lipid levels are not at recommended targets per the CCS guidelines and repeated BP readings indicate mild to moderate hypertension.
**Osteoarthritis**	A 41 year old female began to have right hip pain four years ago that progressed to both hips and knees. Three months of acetaminophen was ineffective for pain relief; naproxen caused dyspepsia and a positive occult blood stool sample. Her smoking cessation efforts (pack-a-day) resulted in weight gain that exacerbated her joint pain. Pain is now daily and may force a job change if not controlled.
**Osteoporosis**	A 75 year old male has become progressively more stooped and experienced episodes of back pain over the past 6 months. Densiometry revealed a BMI > 2 SD below the mean for young men and a serum testosterone level on the low end of normal. He has now suffered a fractured wrist in an accidental fall.

In the osteoarthritis scenario the clinical history suggested that gastrointestinal protection should be considered in the event a non-steroidal anti-inflammatory drug was chosen. Coxibs, an LU product, were a possible choice but there were safety and efficacy data in the public domain that was stimulating debate about their place in therapy. Proton pump inhibitors, in combination with traditional non-steroidal anti-inflammatory drugs, were also recommended by existing guidelines and these were also agents that required an LU prescription for ODBP beneficiaries. The osteoarthritis scenario was included in each focus group session because our earlier research[14] confirmed that general practitioners made different osteoarthritis management choices (3 different prescribing behavior types) depending on the patient's insurance status. However, the medications actually chosen by the participants in response to each scenario were not the focus of the research and were relevant only in so far as they stimulated discussion among the participants on prescribing choice and attitudes towards prescribing.

### Analysis

Focus group recordings were transcribed and verified against the original recordings. A set of preliminary coding categories based on the key questions and content of the data collected were created and then refined and sorted into sub-themes with the assistance of NVivo software. Two researchers independently coded the transcripts and discussed their emergent themes to reach consensus. Content validity was supported through the emergence of substantively similar perspectives held by physicians across the focus groups. Using these themes we describe the common steps of the decision-making process and factors influencing prescribing behavior. It should be noted that focus group responses constitute self-reports and may not predict actual physician behavior in patient consultations.

## Results

### Influence of reimbursement guidelines on prescribing

Physicians reported that during the clinical consult they diagnose and assess concomitant conditions such as social and environmental factors, prior treatment history, risks for adverse events including comorbidity and, if drug therapy is chosen, possible drug interactions. Most physicians in this study, unlike their counterparts elsewhere, explained that they review with patients their ability to pay for medications [15-17]. When most physicians discovered that a patient was over the age of 65 years, received professional services under the home care program, or received social assistance, prescribing decisions based on the reimbursement guidelines of the ODBP came into play.

### Prescribing General Benefit (GB) Drugs

Physicians noted that GB drugs are generally well tolerated and effective. However, some participants perceived that current evidence and clinical experience may run contrary to ODBP guidelines in which multiple-source GB drugs are classified as interchangeable. Participants noted that while GB drugs are commonly viewed as un-restricted, they are not always "hassle-free", and require formulary consultation for dosage compliance and special forms if a "non-substitution" order is required.

Some physicians reported that, despite it not being their first choice, they might still prescribe a GB drug for a variety of reasons: a) the GB drug is covered, thus the patient could get medication immediately, b) the patient's symptoms were not acute at the time of presentation, c) a trial of the GB drug might be effective in improving the patient's condition, d) the GB drug, while not the most effective drug in the physician's opinion, was safe and effective and did no harm, e) the desire to balance quality of care with fiscal restraint, f) if the patient experienced side-effects or the GB drug proved ineffective then the patient's chart would contain the necessary proof that a GB drug was tried and the patient could then meet the LU coverage criteria, and g) the GB drug was a stopgap to be used while awaiting the lengthy Section 8 approval process.

### Prescribing Limited Use (LU) Drugs

Physicians see their primary responsibility - providing the best possible care for patients - as "covenantal"[18]. They find it worrisome when patients do not precisely fit the LU eligibility criteria, especially when clinical experience, interpretation of the evidence, and patient history make a convincing case for an LU drug. One physician explained that, *"it's almost clinical malpractice to give him [the patient] the drug that's free, knowing there is no efficacy, OK?" *Physicians see themselves as duty-bound to fulfill their primary responsibility to patients, but realize that health care costs are rising and that potential legal and financial penalties loom if they are in contravention of ODBP regulations.

### Individual Clinical Review (Section 8) mechanisms and prescribing

"Section 8" refers to the "Individual Clinical Review Mechanisms" section of the ODBP Formulary. It is used when ODBP-eligible patients either may benefit from LU drugs but do not fit the criteria or when they require drugs not listed as GB or LU. Physicians must send a written request for Section 8 review to the Drug Programs Branch of the MOHLTC. Ministry staff coordinates a review that includes recommendations from the DQTC and expert medical advisors. Written requests must provide a diagnosis and rationale for the drug, its trade name, strength and dosage, evidence of effectiveness if the patient has already taken the product, details of alternatives tried including dosages, length of therapy and patient response. Concomitant drug therapy and other relevant information, such as sensitivity reports or laboratory results, are also required[19].

A decision to access Section 8 represents a commitment to writing multiple letters annually to maintain coverage as extensions are not automatic. In cases where the coverage is approved but the dosage changes, a new request must be completed. Some physicians reported that it could take up to four attempts to get a drug covered. It is relatively common to be turned down on the first try.

### Advocating for ODBP-eligible patients

We identified three themes associated with physician advocacy: issues related to time, physician frustration and burnout, and physician-patient rapport. The dominant theme relates to issues of time.

### Issues related to time

Physicians believe that already they do not have enough time with patients and coping with ODBP regulations aggravates this problem. One physician noted, *"I don't book my patients that tight together. I give them their 15 minutes. But my goodness, you know, I consider that my 15 minutes *[are] *for diagnosis and a treatment plan, not to handle bureaucracy and it gets really frustrating." *Other time issues include: a) time to review patient charts for reimbursement eligibility, b) time to interpret the ODBP guidelines and write compliant prescriptions, c) non-billable time for telephone calls with pharmacists, dealing with reimbursement problems or writing Section 8 letters, and d) time spent waiting for Section 8 approval. There is the perception that the prescribing process is deliberately made complex and time-consuming to ensure guideline compliance and that this interferes with timely delivery of quality healthcare.

Physicians noted that they must use non-billable time (usually at the end of the day) to write and fax paperwork. *"Now they *[ODBP]*don't have a 1-800 number if you want to fax so you have to call and wait in line at your own expense for the patient or you have to send it by registered mail as if you have nothing better to do then spend time, at least half an hour, to compose a letter, do it right for the government and then at our own expense, getting it there and wait for rejection and you know, there are a lot of barriers." *Some physicians also perceived an expectation that they complete LU prescriptions and Section 8 letters for specialists that their patients also visit.

### Frustration with the ODBP Program

Physicians generally perceived ODBP guidelines as disincentive hoops, and saw themselves being used as free watchdog gate-keepers to monitor drug costs. They thought that strict adherence to ODBP guidelines might cut drug costs, but could create ancillary costs through poor use of physicians' time and burdening other sectors of the system (i.e., emergency room visits). Most physicians perceive the formulary as complicating rather than facilitating clinical decisions. Words such as "red tape", "hoops", "barriers", and "bureaucracy" were commonly used to describe their experience with the ODBP. In addition, it was common to hear physicians note that they were not confident in the formulary's ability to reflect current evidence or clinical experience.

### Physician "burn-out"

In general, physicians perceived that the ODBP processes seem to have been created to wear down family physicians. They identified the cumbersome 500-page formulary binder, rigid dosages, changing LU codes, complex regulations and long waits for approval (three weeks to three months) as daily hurdles on the ODBP obstacle course. While most physicians reported that they continued to advocate for patients amid frustrations, for some, the daily burden of coping with ODBP barriers resulted in them becoming disheartened and taking the path of least resistance. These physicians could be termed "discouraged advocates"; they surrender and prescribe the GB drug. Physicians reported that they may want to comply with the stepped care approach, (i.e., trying a GB drug and then following all steps needed to access restricted drugs in the event of treatment failure), but they have many patients requiring advocacy and thus are spending more time "jumping through hoops" than providing quality care. *"The frustrating thing, which I think all of us could probably attest to, is that we're the ones always holding the bag, because in the end, patients get all kinds of red tape but the family physician has to wade through all of it." *Some physicians reported that the ministry's inconsistent application of Section 8 guidelines eroded their confidence in the system and their ability to successfully advocate for patients.

### Physician/Patient Rapport

Despite these obstacles, physicians say they advocate because they care about their patients and feel that they are morally and legally bound to provide them with the best care. One physician summed up the common thread heard from most physicians: *"We could say, 'no, sorry, there is nothing that I can do,' but we care." *Physicians admit that the rapport that grows between patients and physicians can influence their decision to go the extra distance, but it is not a requirement. Having patients who appreciate their advocacy encourages physicians to continue to do so. Committing to a patient's case is time consuming over the long and short haul, and requires tenacity and a conviction of the restricted-access drug's efficacy.

Factors that influence family physicians' decisions not to advocate fall into two categories relating to patients' attributes and the working conditions and policies associated with practicing medicine. Physicians noted that they tend to advocate more actively for patients who do not have the income to pay for non-covered drugs. They are disinclined to advocate for patients who have a strong a sense of entitlement even when they can pay or those who pay for expensive alternative/complementary therapies of dubious benefit but resist paying for a more effective but non-covered medication.

Some physicians reported that they typically avoid consulting the ODBP formulary when writing prescriptions except in extreme circumstances. The most common reason given is that the formulary was not usually in the examination room and/or referring to it is onerous and consumes precious consultation time with their patients. Some physicians explained that they write prescriptions for drugs and let the pharmacist research, screen and sort out the ramifications regarding ODBP coverage. The general trend in responses from these physicians is that they see their responsibility as primarily caring for their patients rather than implementing cost-containment strategies on behalf of the provincial government. In situations where a patient's need was acute, these discouraged advocates would advocate for the patient to receive coverage, but they appear to struggle with taking a proactive stance.

## Discussion

Formulary-based public drug plans extend health coverage for most financially disadvantaged populations and, in some Canadian provinces, for their citizens as a whole. The formulary review process that is part of every public drug plan in Canada assesses each new drug for safety, efficacy, and cost-effectiveness. This provides physicians with some assurances of the composite value of those drugs that do get listed on the formulary. While physicians in this study generally agreed that cost-effectiveness is a worthy goal, they did not generally see their role as cost monitors. With respect to their experience with the ODBP as it was structured in 2002, they felt it would be more effective if it more accurately reflected standard clinical practice and current clinical guidelines and was less complicated to administer. Physicians also argued that, while the ODBP should ideally contribute to the improvement of the quality of care and keep costs down, it can also contribute to lower quality of care and to the misuse of physicians' time thereby increasing costs to the publicly-funded health care system in Ontario by adding unnecessary visits to family physicians, specialists, and emergency rooms.

Data from this relatively small qualitative study suggests that advocacy is situationally bound. While we learned that physicians' decisions to advocate are influenced by their understanding of the patient's condition, the level and immediacy of risk created by the patient's condition, and the perceived benefit of a restricted drug, we discovered that their decision to advocate is also influenced by having the necessary time, stamina, conviction, and encouragement. These findings are similar to those of other published literature[20,21]. Findings reveal that the ODBP regulations of 2002 were seen by these physicians as complex and onerous and had a substantial impact on the physician-patient relationship and on patient outcomes. The findings and conclusions of this preliminary study also find resonance in the Ontario Medical Association's response to the Drug Strategy Review (DSR) that preceded the recent reforms to the ODBP: "When considering the LU and Section 8 processes the DSR Steering Committee should not underestimate the depth and intensity of physician enmity to these programs"[22]. When reimbursement hurdles remain difficult and numerous, there will be some physicians who will become worn down. The result is that reimbursement guidelines can influence medication choice and can produce unequal access to medication.

## Conclusion

This study provides an understanding of family physicians' attitudes about prescribing under the ODBP formulary as it was designed in 2002. At that time, these family physicians were concerned about the impact of the ODBP formulary on patient outcomes, the loss of their diagnosis and treatment time due to drug coverage paperwork, whether some generic drugs were as effective as name-brand counterparts, and the lengthy wait times for drug coverage that they felt could put some patients at risk. These concerns were addressed in 2006 when the provincial government passed legislation to overhaul the ODBP to increase overall transparency and accountability, establish faster drug funding decisions, assess the administrative barriers of the Section 8 mechanism, and replace it with a new faster process with the intention of dramatically reducing paperwork for physicians and pharmacists[23].

Allan and Innes report that Canadian physicians in British Columbia have limited knowledge of the actual price of drugs[24]. However, physicians do know that if a drug is not included in a formulary it is likely to be more costly than formulary alternatives[24]. It may be that the onerous nature of the old ODBP guidelines actually precipitated physicians asking about coverage and ability to pay because they were attempting to determine if they would require additional time to complete paperwork. Alternatively, physicians could be responding to a social desirability factor.

While the focus of this qualitative study was not family physicians' knowledge of drug costs but on their attitudes about prescribing using a formulary, we learned that these family physicians' knowledge of relative costs of drugs is based on their experience that formularies list less costly generic or brand-name drugs and not newer more expensive ones. Family physicians reported that they learned the costs of drugs through daily clinical practice, especially when their patients returned to report the high price of a drug they prescribed or when pharmacists called saying their patients weren't covered or couldn't afford a drug. Reports of formulary inclusion decisions indicate that the coverage decision can be sensitive to the price of a medication [25,26]. In their analysis of 58 drugs approved for sale in Canada in 1996-1997, Anis et al[25] found a significant association (p < .001) between the price ratio (price of the new drug relative to price of the cheapest available comparator on the formulary) and whether coverage was granted.

### Study Limitations and directions for future research

The findings from this preliminary study cannot be generalized to physicians practicing in other provinces or countries, or Ontario physicians practicing under the new ODBP regulations. Our findings differ substantially from US data regarding the practice of family physicians asking patients about their ability to pay. It may be that administrative hurdles actually precipitate physicians asking and is an attempt to gauge the impact of embarking on patient advocacy in relation to the themes described in this study. The relative influence of a social desirability factor could not be determined from our results. Moreover, it is possible that some physicians in this study lacked full and accurate information in some aspects of the ODBP, and this could have influenced their discussion. Nonetheless, their comments are still valid, as they are making prescribing decisions for their patients with this limited understanding. Future research, possibly with a larger sample using a survey instrument designed around the themes uncovered in this study, might prove valuable. In addition, Allen, Lexchin and Wiebe argue that understanding the accessibility and reliability of medical cost information provided to physicians and whether physicians use that information and prescribe differently as a result are important topics to be explored[27].

It emerged from this study that physicians have concerns about the impact of drug substitution or delays in obtaining drugs on patient outcomes, and more research is required to explore these concerns. Given the administrative reforms recently enacted in the ODBP, it would be worthwhile to replicate and enlarge on this research to examine whether or not physicians' attitudes and prescribing practices have changed especially in light of new ODBP changes. Applying the structured inquiry described here to the new prescribing context in order to compare and contrast results would provide a rare opportunity to measure the impact of specific aspects of drug plan policy.

## Competing interests

Dr. Susan Grant and Mr. Kevin Skilton were, at the time of the study, members of the Corporate Affairs Group of Merck FrosstCanada and were engaged in Canadianpublicpolicy respecting access to innovative medicines. They participated in the development ofthe research question, but not in the analysis of the results. Dr. Grant developed the scenarios, assisted in transcription of the medical terminology, and in refining the presentation of the analysis of physician prescribing behaviour. All publications reporting research in which Merck Frosst participatesare reviewed prior to publication in accordance with corporate disclosure policy.

This research was sponsored by Merck Frosst, and performed by McMaster University researchers and staff. The full study team confirms that this paper reflects our own interpretation of the data and that Merck Frosst has not biased the research in any way.

## Authors' contributions

All authors participated in the study design and coordination and helped to draft the manuscript. All authors read and approved the final manuscript.

## Pre-publication history

The pre-publication history for this paper can be accessed here:


